# Early pregnancy body mass index and gestational weight gain: A mediating or moderating factor for short stature and risk of gestational diabetes mellitus?

**DOI:** 10.1371/journal.pone.0272253

**Published:** 2022-08-01

**Authors:** Heng Yaw Yong, Zalilah Mohd Shariff, Barakatun Nisak Mohd Yusof, Zulida Rejali, Yvonne Yee Siang Tee, Jacques Bindels, Eline M. van der Beek

**Affiliations:** 1 Department of Nutrition, Faculty of Medicine and Health Sciences, Universiti Putra Malaysia, Selangor, Malaysia; 2 Department of Dietetics, Faculty of Medicine and Health Sciences, Universiti Putra Malaysia, Selangor, Malaysia; 3 Department of Obstetrics and Gynaecology, Faculty of Medicine and Health Sciences, Universiti Putra Malaysia, Selangor, Malaysia; 4 Danone Specialized Nutrition (Malaysia) Sdn. Bhd., Kuala Lumpur, Malaysia; 5 Research Foundation, Nieuwvee, The Netherlands; 6 Department of Pediatrics, University Medical Centre Groningen, University of Groningen, Groningen, The Netherlands; Tabriz University of Medical Sciences, ISLAMIC REPUBLIC OF IRAN

## Abstract

This study examined the association between height and the risk of Gestational Diabetes Mellitus (GDM), and whether this association was mediated or moderated by early pregnancy body mass index (BMI) and gestational weight gain (GWG) that are known independent risk factors for GDM. Data of a retrospective cohort of pregnant women (N = 1,945) were extracted from antenatal clinic cards. The cut-off values of height in relation to risk of GDM were identified using receiver operating characteristic analysis and four categories of height were derived: < 150 cm, 150–155 cm, 156–160 cm, and > 160cm. Mediation analysis was performed using the Preacher and Hayes bootstrapping method while the moderation effect was tested with multiple regression analysis with interaction terms. Although there was no mediation effect of BMI and GWG on the association between height and risk of GDM, both factors moderated this association with a significant association between shorter height and risk of GDM was observed in overweight / obese women (height < 150 cm: AOR = 1.41, 95% CI = 1.03–2.44; height 156–160 cm: AOR = 1.48, 95% CI = 1.03–2.14). Overweight / obese women with height < 150 cm and excessive GWG at the end of the second trimester (AOR = 2.25, 95% CI = 1.45–4.17) had significantly higher risk of GDM than those without these factors. Short stature (< 150 cm) was significantly associated with GDM risk among OW/OB women with excessive gestational weight gain at the end of second trimester. This finding underscores the importance of maintaining a healthy BMI during reproductive age and gaining weight in recommended range during pregnancy.

## Introduction

Brown et al. (1991) was the first to report that adult height was negatively associated with glucose tolerance [1]. Several studies further confirmed this observation in the general population, which showed that type 2 diabetes mellitus (T2DM) [2, 3] was more prevalent in adults with short stature. Similarly, among women, those with short stature were significantly at a higher risk for T2DM and gestational diabetes mellitus (GDM) [4–8]. However, the threshold for defining short maternal stature that is associated with GDM risk seemed to vary across populations, ranging from 151 cm to 160 cm [4–6, 8].

Studies on the relationship between maternal height and risk of GDM are limited and with conflicting findings. While several studies found no significant relationship between height and GDM risk [9, 10], others showed that maternal height was negatively related to risk of GDM [5, 6, 11–13]. Similarly, a recent systematic review and meta-analysis reported that each 5 cm increase in height was related with a 20% decrease in GDM risk, and the relationship did not differ significantly between Asian and non-Asian women [14]. In the general population, short stature has been shown to be associated with the risk of overweight and obesity in adults and children [15, 16]. It has also been suggested that in pregnant women, short stature when combined with body mass index (BMI) greater than 25, may pose an additional risk for GDM [5, 17]. It is unclear if the association between short stature and GDM is directly or indirectly influenced by pre-pregnancy BMI and GWG. However, the relationship between height and GDM and the possible interaction of the association with other factors (mediating or moderating), is worthwhile to be investigated particularly in the context of developing countries.

Little is known of the mechanisms by which height or maternal stature may affect blood glucose level. It is uncertain if the association between short stature and glucose level is biologically meaningful or simply artefactual. Since GDM is commonly diagnosed based on glycemic response to a standard oral glucose load, the relationship between short stature and GDM may be due to the fact that the 75g OGTT test represents a greater glycemic stimulus in shorter women [12, 18]. For instance, shorter women may have a lower mass of metabolically active tissues to respond to a standard OGTT compared to taller women. Hence, this may also explain why Asian women on average who are shorter than European women, are more likely to be diagnosed with GDM. Height is also a reflection of the quality of environment whereby women of lower social class are generally shorter than those of higher social class [19]. Women with short stature could have experienced undernutrition not only during childhood but also during the present time [20, 21]. Poor nutrient but high energy-dense diets, especially mixtures of sugars and fats, that are relatively inexpensive and affordable to the lower social class women maybe a plausible explanation for the association between height and the risk of GDM [22]. It is also possible that a certain gene, which defines height, may influence the susceptibility to glucose intolerance [11]. For instance, a polymorphism in the gene for insulin-like growth factor-1 (IGF-1) functional properties has been shown to be related to short stature and subsequent increased risk for GDM [18].

The prevalence of GDM is rising worldwide [23], with the GDM rate in the Malaysian population [24] deemed to be higher than the reported rates in other Asian countries [25, 26]. In Malaysia, height is not considered as a risk factor for GDM in screening of pregnant women at clinics or hospitals but maternal height (less than 145 cm) has been included in the selection criteria for hospital birth or home birth [34]. Since hormonal and nutritional experiences are major risk factors of short stature, maternal height may be a potential risk factor of GDM in the Malaysian population. To date, studies on the association between height and risk of GDM are restricted to Western population [4, 5, 12, 27, 28] with few studies in the Asian population [6, 8]. Furthermore, adult height has changed over the past century, with the amount of change varied across countries [29]. It is worthwhile to explore the association of height of women in the 20^th^ century and GDM risk, particularly in countries where short stature and diabetes mellitus are prevalent. Although it is well recognized that both obesity and excessive gestational weight gain (GWG) confer higher risk of GDM, previous studies did not consider pre-pregnancy BMI or GWG as potential factors interacting with the association between maternal height and GDM. Therefore, this study aimed to determine the relationship between maternal height and risk of GDM and to explore the roles of BMI and GWG as mediators (direct / indirect) or moderators to better understand the association between height and GDM.

## Materials and methods

### Study design and location

This was a retrospective cohort study of healthy pregnant women with a singleton gestation attending antenatal care at two randomly selected government health clinics in the urban district of Seremban, located in a southern state (Negeri Sembilan) of Peninsular Malaysia. A total of 2,209 of pregnant women were initially identified for potential inclusion in this study. The details of the study protocol have been described previously [30]. Two hundred and fifty-eight women were subsequently excluded from the analysis, as they were < 18 years old (n = 16) or had abnormal glycemia (n = 242). In addition, 6 women with extreme height value (< 135cm, > 180cm) were excluded. The final sample size was 1,945 pregnant women.

Data source was antenatal clinic cards of pregnant women. The antenatal clinic card consists of four main sections, i.e. demographic and obstetric information, anthropometric measurements, biochemical data and birth information. All data were extracted by trained enumerators. The study protocol was approved by the Medical Research Ethics Committee (MREC), Universiti Putra Malaysia (UPM/FPSK/100-9/2-MJKEtika) and the Medical Research Ethics Committee (MREC), Ministry of Health Malaysia (KKM/NIHSEC/08/0804/P12-613). As the study was retrospective in nature, informed consent was not required, and anonymity of all participants was retained before and during data analysis.

### Anthropometric measurements

Height in centimetres (cm) at the first prenatal visit was obtained from the antenatal cards. The receiver operating characteristic (ROC) was used to determine a cut-off value for height, below which the GDM risk substantially increased. The ROC showed that the calculated cut-off value was 160 cm, with an area under the curve (AUC) of 0.531 (p< 0.05). A 145 cm cut-off value is used for the diagnosis of short stature in the Malaysian population [31]. However, as only 1.4% of women in this study were with height less than 145 cm, the lowest cut-off value for height in this study was set at < 150 cm. Four categories of height were derived (< 150 cm, 150–155 cm, 156–160 cm, and > 160cm) with the highest category (> 160 cm) was used as the reference group in the multivariate analysis.

Weight at first prenatal visit, first trimester and second trimester were obtained from the antenatal clinic cards. Pre-pregnancy body mass index (BMI) was estimated using weight at first prenatal visit as there was no pre-pregnancy body weight data in the antenatal cards. The BMI was determined by dividing the weight (kilogram) by the square of height at the first prenatal visit (meter2), and subsequently categorized into four groups: < 18.5 kg/m^2^ for underweight, 18.5–24.9 kg/m^2^ for normal weight, 25.0–29.9 kg/m^2^ for overweight, and ≥ 30.0 kg/m^2^ for obese [32]. The difference between the measured weight at the first prenatal visit and the last clinically recorded weight at the end of the second trimester (28^th^ weeks of gestation) was defined as total GWG at the end of the second trimester. The rate of GWG at second trimester was defined as the average weekly weight increase throughout the second trimester. Using the US Institute of Medicine (IOM) recommendations (2009), total GWG at the end of the second trimester and rate of GWG at the end of the second trimester were classified as inadequate, adequate, or excessive for each pre-pregnancy BMI category. Inadequate GWG and excessive GWG were defined as weight gain below and above the recommended guideline.

### Maternal glucose level

Between the 28th and 32nd week of gestation, all pregnant women were required to undergo a standard 2-hour 75g oral glucose tolerance test (OGTT) [31]. GDM was diagnosed if either or both fasting plasma glucose (FPG) was ≥ 5.6 mmol/L or 2-hours plasma glucose (2hPG) is ≥ 7.8mmol/L [31].

### Demographic characteristics and obstetric history

Maternal demographic and obstetric information were obtained from the antenatal clinic cards. The information included ethnicity (Malay versus non-Malay), age (< 35 versus ≥ 35 years old), occupation (housewife versus others), education (lower-secondary versus others), and parity (0, 1–2, and ≥ 3).

### Statistical analysis

All analyses were carried out using the IBM SPSS Statistics version 23 with the exception of the mediation analyses that used the SPSS PROCESS macro [33]. Exploratory Data Analysis (EDA) was performed to determine the normality and homogeneity of the data. There was no data transformation as all continuous variables were normally distributed. Means and standard deviations were reported for continuous variables while frequency and percentage distributions for categorical variables. For continuous and categorical variables, the Chi-square test of independence or Fisher’s exact test and independent t-test were employed to examine the relationship between women’s characteristics and the risk of GDM.

Binary logistic regression (enter method) was used to examine the association between height in both continuous and categorical forms with GDM risk. Age and BMI at first prenatal visit, total GWG at the end of second trimester, and gestational week at OGTT performed (all in continuous form) were included as covariates in the multivariable model. Moderation effects of GWG (continuous) and BMI at first prenatal visit (continuous) were examined using logistic regression by including interaction terms. Stratified analyses were performed for all significant interaction terms. Adjusted odds ratio (AOR) with 95% confidence interval (CI) were presented. A significant level of the statistical analysis was set at p< 0.05.

Mediation analyses to determine the role of GWG and BMI as potential mediators of the association between height and GDM risk were performed using the Preacher and Hayes bootstrapping method [33]. Direct effect of height on GDM risk and the indirect effects of mediators on the association between height and GDM were estimated. Two models per outcome were performed to test the specific effects of GWG (rate of GWG at second trimester or total GWG at the end of second trimester) and BMI at first prenatal visit, separately and together, adjusted for age and the gestational week at OGTT performed.

## Results

Table 1 shows the characteristics of GDM and non-GDM women. The prevalence of GDM in this study population was 13.1%. Non-GDM (28.89 ± 4.36 years old) women were significantly younger than GDM women (30.30 ± 4.77 years old, p< 0.001). There were no significant differences in ethnicity, education level, occupation status, and parity between non-GDM and GDM women (p> 0.05). For anthropometric measurements, non-GDM women were significantly taller (156.15 ± 5.66 cm), had lower weight at the first prenatal visit (60.07 ± 14.14 kg), but had higher total GWG at the end of the second trimester (5.98 ± 3.44 kg) compared to GDM women (155.38 ± 5.53 cm; 62.31 ± 13.49 kg; 5.40 ± 3.56 kg, p < 0.05).

**Table 1:** Characteristics of women with GDM and non-GDM (N = 1945).

	Maternal glycemia^a^		p-value
	Non-GDM (n = 1691)	GDM (n = 254)	
Age (years old) (*M* ± *SD*)	28.89 ± 4.36	30.30 ± 4.77	0.001**
• < 35	1516 (89.7)	203 (79.9)	0.001**
• ≥ 35	175 (10.3)	51 (20.1)	
Ethnicity			
• Malay	1425 (84.3)	205 (80.7)	0.15
• Non-Malay	266 (15.7)	49 (19.3)	
Education level			
• Lower-secondary	1007 (59.6)	155 (61.0)	0.66
• Others	684 (40.4)	99 (39.0)	
Occupation			
• Housewife	659 (39.0)	100 (39.4)	0.90
• Others	1032 (61.0)	154 (60.6)	
Parity (*M* ± *SD*)	1.41 ± 1.28	1.57 ± 1.46	0.07
• 0	483 (28.6)	69 (27.2)	0.31
• 1–2	459 (27.1)	60 (23.6)	
• ≥ 3	749 (44.3)	125 (49.2)	
Height (cm) (*M* ± *SD*)	156.15 ± 5.66	155.38 ± 5.53	0.04*
• < 150	195 (11.5)	36 (14.2)	0.07
• 150–155	566 (33.5)	82 (32.3)	
• 156–160	573 (33.9)	98 (38.6)	
• > 160	357 (21.1)	38 (15.0)	
Weight at the first prenatal visit (kg) (*M* ± *SD*)	60.07 ± 14.14	62.31 ± 13.49	0.02*
BMI at the first prenatal visit (kg/m^2^) (*M* ± *SD*)	24.62 ± 5.52	25.74 ± 5.12	0.001**
• Underweight (< 18.50)	187 (11.1)	19 (7.5)	0.01*
• Normal (18.50–24.99)	792 (46.8)	101 (39.8)	
• Overweight (25.00–29.99)	468 (27.7)	87 (34.3)	
• Obese (≥ 30.00)	244 (14.4)	47 (18.4)	
Rate of GWG at the second trimester (kg/week) (*M* ± *SD*)	0.41 ± 0.01	0.39 ± 0.01	0.14
• Inadequate	526 (31.1)	83 (32.7)	0.43
• Adequate	444 (26.3)	57 (22.4)	
• Excessive	721 (42.6)	114 (44.9)	
Total GWG at the end of second trimester (kg)	5.98 ± 3.44	5.40 ± 3.56	0.01*
• Inadequate	1012 (59.8)	146 (57.5)	0.77
• Adequate	461 (27.3)	74 (29.1)	
• Excessive	218 (12.9)	34 (13.4)	
Maternal glucose level (OGTT) (mmol/l) (*M* ± *SD*)			
Gestational age at OGTT performed (weeks)	28.39 ± 1.45	28.46 ± 1.63	0.49
Fasting plasma glucose (FPG)	4.29 ± 0.41	4.90 ± 0.73	0.001**
2-hours plasma glucose (2hPG)	5.75 ± 1.05	8.40 ± 1.35	0.001**

Abbreviations: BMI, body mass index; OGTT, Oral glucose tolerance test

Note.

^a^ GDM was classified according to MOH criteria, either FPG ≥ 5.6mmol/l or 2hPG ≥ 7.8 mmol/l or both.

*p<0.05

**p<0.001

**Fig 1:**
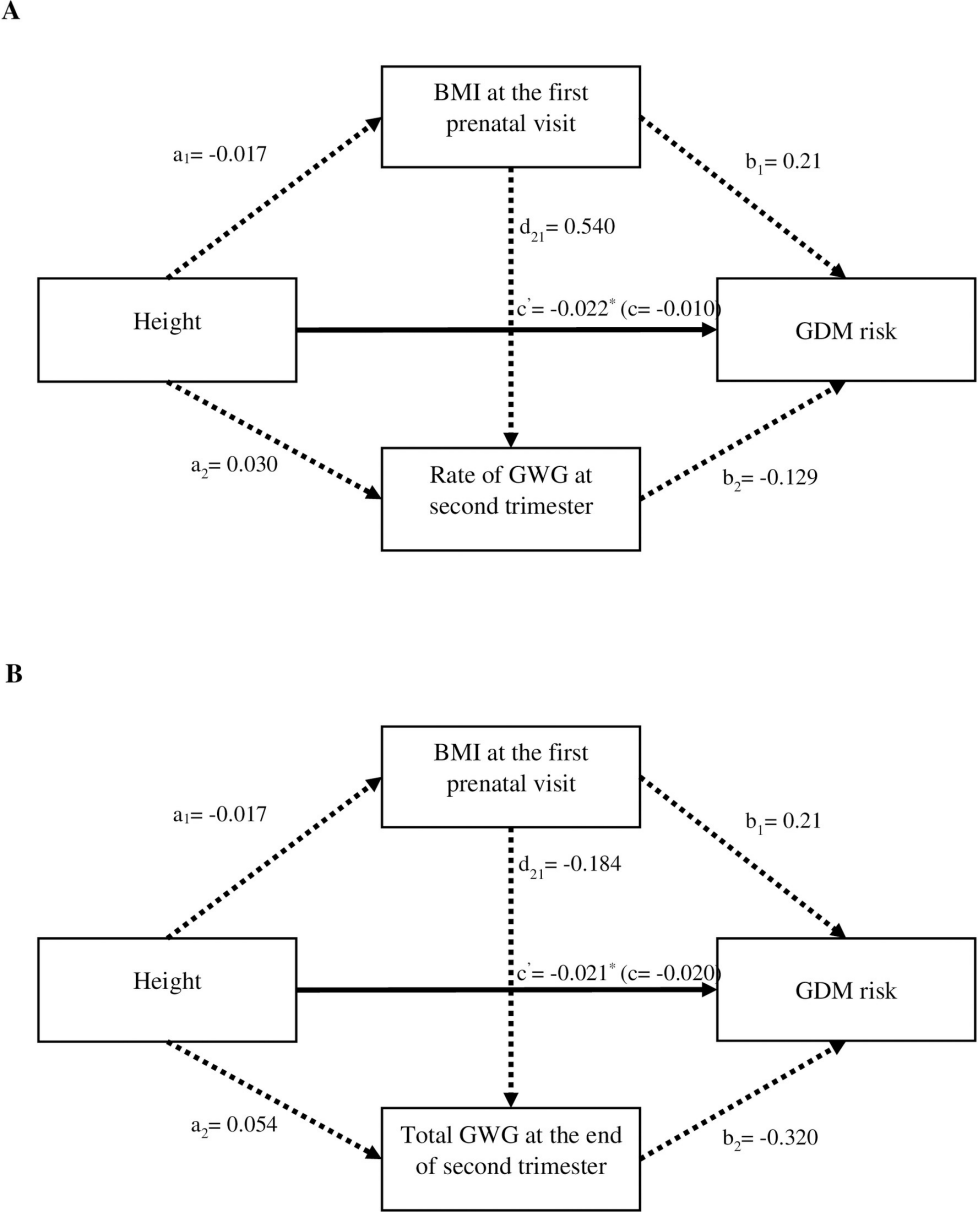
The association between height and GDM risk mediated by BMI at the first prenatal visit and GWG. Bold lines indicate significance of the effect at p< 0.05. Dashed lines indicate that the effects are not significant (p> 0.05). Note. ^A & B^ Adjusted for age and gestational week at OGTT performed.

Fig 1 and Table 2 present the mediation analysis for the association between height and GDM risk as mediated by BMI at the first prenatal visit and/or GWG. The findings did not support the hypothesis that the association between height and the risk of GDM is mediated by BMI and/ or GWG. Table 3 shows the associations between height and GDM risk. Height, respectively for continuous (AOR = 0.97, 95% CI = 0.95–0.99) and categorical variables were significantly associated with the risk of GDM. This significant association was observed in overweight / obese (OW/OB) women, whereby OW/OB women with height less than 150 cm (AOR = 1.41, 95% CI = 1.03–2.44) or height between 156 and 160 cm (AOR = 1.48, 95% CI = 1.03–2.14) had significantly higher risk for GDM compared to underweight / normal weight (UW/NW) women (p< 0.05). Although not significant, the AOR for women with height 150–155 cm shows similar direction of GDM risk (Table 4). The stratified analyses by BMI and GWG categories (Table 5) further suggest that women with height < 150 cm, OW/OB, and had excessive GWG at the end of the second trimester were significantly at higher risk for developing GDM than those without these factors.

**Table 2:** Indirect associations between height and the risk of GDM by mediators.

	Coeff.	Boost SE	Bootstrapped 95% bias correct CI	
			Lower	Upper
(A) Indirect effect (Path ab)^a^				
• Ind 1 (a_1_b_1_)	-0.004	0.007	-0.020	0.008
• Ind 2 (a_2_b_2_)	-0.004	0.011	-0.028	0.018
• Ind 3 (a_1_d_21_b_2_)	0.001	0.011	-0.028	0.017
(B) Indirect effect (Path ab)^b^				
• Ind 4 (a_1_b_1_)	-0.004	0.007	-0.020	0.008
• Ind 5 (a_2_b_2_)	-0.017	0.013	-0.044	0.006
• Ind 6 (a_1_d_21_b_2_)	-0.001	0.002	-0.005	0.002

Note. CI–confidence interval, BMI at the first prenatal visit–body mass index at the first prenatal visit, GWG–gestational weight gain

(A) Ind 1 –Maternal height → BMI at the first prenatal visit → GDM; Ind 2 –Maternal height → rate of GWG at the second trimester → GDM; Ind 3 –Maternal height → BMI at the first prenatal visit → rate of GWG at the second trimester → GDM

(B) Ind 4 –Maternal height → BMI at the first prenatal visit → GDM; Ind 5 –Maternal height → total GWG at the end of second trimester → GDM; Ind 6 –Maternal height → BMI at the first prenatal visit → GWG at the end of second trimester → GDM

^a,b^ Adjusted for age and gestational week at OGTT performed

If zero does not occur between the lower level and upper level CI, the indirect effect for this mediator is deemed to be significant.

**Table 3:** Adjusted odds ratio (OR) and 95% confidence intervals (95% CI) for associations between height and gestational diabetes mellitus (GDM) (n = 1945).

	Wald	p-value	GDM
			AOR [95% CI]
Height ^a^	7.32	0.04	
• < 150			1.65 [1.01–2.70]*
• 150–155			1.28 [0.85–1.93]
• 156–160			1.62 [1.09–2.42]*
• > 160			1.00
Interaction terms ^b^			
Height x BMI at first prenatal visit ^c^	12.70	0.01	
• < 150			1.02 [1.01–1.04]*
• 150–155			1.01 [0.99–1.02]
• 156–160			1.02 [1.01–1.04]**
• > 160			1.00
Height x rate of GWG at second trimester ^d^	6.65	0.08	
• < 150			1.77 [0.69–4.49]
• 150–155			0.89 [0.44–1.80]
• 156–160			1.88 [0.99–3.38]
• > 160			1.00
Height x total GWG at the end of second trimester ^e^	2.34	0.51	
• < 150			1.03 [0.97–1.10]
• 150–155			0.98 [0.94–1.03]
• 156–160			1.03 [0.99–1.07]
• > 160			1.00

^a^ Adjusted by age, BMI at first prenatal visit, total GWG at the end of second trimester, and gestational week at OGTT performed

^b^ Interaction terms: BMI at first prenatal visit, rate of GWG at second trimester, and total GWG at the end of second trimester.

^c^ Adjusted for age, total GWG at the end of second trimester and gestational week at OGTT performed.

^d^ Adjusted for age, BMI at first prenatal visit and gestational week at OGTT performed.

^e^ Adjusted for age, BMI at first prenatal visit and gestational week at OGTT performed.

*p<0.05

**p<0.01

**Table 4:** Adjusted odds ratio (OR) and 95% confidence intervals (95% CI) for the associations between height and gestational diabetes mellitus (GDM) stratified by BMI at prenatal visit.

Height (cm)	BMI status at the first prenatal visit ^a^			
	UW/NW (n = 1099)		OW/OB (n = 846)	
	n	AOR [95% CI]	n	AOR [95% CI]
• < 150	125	1.11 [0.65–1.90]	106	1.41 [1.03–2.44]*
• 150–155	357	0.75 [0.51–1.12]	292	1.29 [0.89–1.87]
• 156–160	396	1.05 [0.74–1.49]	275	1.48 [1.03–2.14]*
• > 160	221	1.00	183	1.00

Noted. AOR = Adjusted OR; 95% CI = 95% confident interval

^a^ Adjusted by age, total GWG at the end of second trimester, and gestational week at OGTT performed

*p< 0.05

**Table 5:** Adjusted odds ratio (OR) and 95% confidence intervals (95% CI) for associations between height and gestational diabetes mellitus (GDM) stratified by BMI at the prenatal visit.

Height (cm)	BMI at the first prenatal visit							
	UW/NW ^a^				OW/OB ^a^			
	Inadequate GWG (n = 779)		Excessive GWG (n = 68)		Inadequate GWG (n = 379)		Excessive GWG (n = 184)	
	n	AOR [95% CI]	n	AOR [95% CI]	n	AOR [95% CI]	n	AOR [95% CI]
• < 150	103	0.83 [0.36–1.92]	3	1.15 [0.31–4.21]	52	0.70 [0.26–1.88]	15	2.25 [1.45–4.17]*
• 150–155	255	1.09 [0.67–1.79]	23	0.95 [0.44–2.04]	149	0.59 [0.18–1.22]	57	1.86 [0.57–2.06]
• 156–160	268	0.75 [0.40–1.40]	29	1.76 [0.33–2.77]	109	0.97 [0.95–1.39]	54	1.55 [0.46–5.25]
• > 160	153	1.00	13	1.00	69	1.00	58	1.00

Noted. AOR = Adjusted OR; 95% CI = 95% confident interval

^a^ Adjusted by age and gestational week at OGTT performed

*p< 0.05

## Discussion

This study further extends the findings of previous studies on the association between height and the risk of GDM by demonstrating that the association may be mediated or modified by BMI and GWG. The observed association between shorter height and GDM risk was not mediated by BMI and GWG, but the significant association was only found among OW/OB women with excessive total GWG at the end of the second trimester. Overall, the present study found that the associations between BMI or GWG with height and GDM risk were considered as weak to moderate. The weak association observed could be the main reason for the mediating role of BMI or GWG was not supported in this study. As the mediation analysis included a wide range of BMI and GWG values, this could explain the non-significant observation for both associations. However, for the moderation analysis, the observations seemed to support that the association of height and GDM risk is specific to those with high body fat, reflected by being overweight/obese and with excessive gestational weight gain prior to GDM diagnosis. Although both overweight/obesity (OV/OB) and excessive gestational weight gain are well-known risk factors for adverse pregnancy outcomes, particularly GDM [30, 34], further studies are needed to elucidate, in depth, these intertwined associations in relation to the development and severity of GDM.

The present study showed that the association between shorter height and risk of GDM was observed only in OW/OB women. Similarly, a study in China [13] found that the association between height and GDM risk was limited to OW/OB women with BMI ≥ 24 kg/m^2^ [8]. A further stratified analysis of BMI at first prenatal visit and GWG at the second trimester revealed that particularly women with height < 150 cm, OW/OB and excessive GWG at the end of the second trimester were significantly at higher risk for developing GDM. The associations between higher pre-pregnancy BMI and excessive GWG with the risk of GDM have not only been established in previous studies [35, 36] but also in previously reported data of a prospective cohort study in Malaysia (SECOST) [30, 37, 38]. It is possible that women with short stature and higher pre-pregnancy BMI have limited muscle and higher fat mass [39]. As women are already in a mild state of insulin resistance during early pregnancy, the excessive GWG in addition to higher pre-pregnancy BMI of women with short stature may further enhance beta cell dysfunction and increase the risk of GDM [40].

The association between shorter height and risk of GDM is consistent with several previous studies [5–7], which reported that maternal short stature was associated with an increased risk of GDM after controlling for covariates. The threshold for defining short maternal stature that increased the risk of developing GDM varied across studies [4–6, 8]. As most of these studies [4–6] derived the cut-off level of height from the data itself (by tertiles, quartiles, quintiles), rather than practical cut-off level for diagnostic or screening purposes, comparison between studies is difficult. However, most studies examining one or more pregnancy outcomes showed that maternal height less than 160 cm was associated with increased risk of adverse pregnancy outcomes [4–6, 8]. Similarly, this study also found that women with height below 160 cm were substantially at increased risk of GDM. More well-designed studies are needed to establish an optimal cut-off for short stature in terms of predicting probability of adverse outcomes.

The present study also found that taller women had significantly lower 2hPG, but not FPG compared to shorter women (S1 Table). This finding is supported by the evidence that in the general population, taller adults were more likely to have lower postprandial glucose levels compared to shorter adults using a standard OGTT test, with no difference in fasting glucose level [39]. Taller adults have more muscle mass, which is the major tissue involved in glucose metabolism [39]. In addition to possible muscle mass effect, the dilution effect of total body water is another plausible explanation [39]. More studies with larger samples are needed to further confirm this association, as well as the suitability of using OGTT to diagnose GDM.

Several study limitations warrant consideration. Several important risk factors of GDM such as family history of T2DM, history of GDM, physical activity level, and dietary habits, were not assessed. The study used standing height and did not consider other components of height, such as trunk length, and leg length due to unavailable data, which might be a better risk factor of GDM. Ma et al.^46^ showed that short leg length was an independent determinant of GDM in the Chinese population. Additionally, weight at first prenatal visit was used to estimate pre-pregnancy BMI and GWG which could introduce estimation error. Nevertheless, Holland et al. (2014) showed that weight at the first prenatal visit and self-reported pre-pregnancy weight yielded similar pre-pregnancy BMI classification [41]. As this study did not examine other adverse pregnancy outcomes such as pre-term birth, low birth weight and small- or large-gestational age, it is unknown whether maternal short stature height could adversely impact pregnancy outcomes or short stature combines with OV/OB and excessive GWG could confer a higher risk of complications in pregnancy. Despite these limitations, this study provides an insight on mediating and moderating effect of BMI and GWG for the relationship between height and GDM risk.

## Conclusions

The present study found that maternal short stature was related with an increased risk of GDM. In specific, this significant association was not mediated by BMI and GWG but modified by the effect of BMI at the first prenatal visit and total GWG at the end of the second trimester. Women with short stature and higher pre-pregnancy BMI might already experience a mild state of insulin resistance during early pregnancy and that having excessive GWG could exert additional burden to the existing beta cell dysfunction which further increased the risk of GDM. As insulin resistance existing before/during pregnancy can be progressive, these women are also at higher risk of developing metabolic syndrome and consequently type 2 diabetes mellitus at later life if no precautions are taken. The relationship between short stature and GDM risk should be further explored, considering other compounding risk factors, such as excessive GWG, family history of T2DM, history of GDM. It is also of importance to address possible intergenerational effects of short stature (e.g., epigenetic effects, shared genetic characteristics, metabolic change programming) on risk of GDM as well as to promote healthy BMI during reproductive age and weight gain within recommended range during pregnancy.

## Supporting information

S1 Table The fasting plasma glucose and 2-hours plasma glucose by height categories. (DOCX)
