# Supplementary material for: Early pregnancy body mass index and gestational weight gain: A mediating or moderating factor for short stature and risk of gestational diabetes mellitus?
Source: PLoS One. 2022 Aug 1;17(8):e0272253. doi: 10.1371/journal.pone.0272253 (PMC9342715; doi:10.1371/journal.pone.0272253)
Supplement: S1 Table — (DOCX) [file pone.0272253.s001.docx]

**Supplementary Table 1. The fasting plasma glucose and 2-hours plasma glucose by height categories**

|  | **Height (cm)** |  |  |  | **p-value** |
| --- | --- | --- | --- | --- | --- |
|  | **< 152** | **152 – 156** | **157 – 160** | **> 160** |  |
| **Fasting plasma glucose, mmol/L** | 4.33 ± 0.53 | 4.39 ± 0.53 | 4.37 ± 0.49 | 4.37 ± 0.46 | 0.26 |
| **2-hours plasma glucose, mmol/L** | 6.21 ± 1.50^a^ | 6.14 ± 1.40^b^ | 6.05 ± 1.37 | 5.96 ± 1.37^a,b^ | 0.04* |

*p< 0.05

^a,b^ Similar letters in superscript indicate significant differences.
